# Massive Puerperal Vulvovaginal Hematoma Following Vaginal Delivery: A Case Report

**DOI:** 10.7759/cureus.108182

**Published:** 2026-05-03

**Authors:** Anibri Mouna, Benabdeslam Rim, Fethi Khalid

**Affiliations:** 1 Department of Obstetrics and Gynecology, Oncology and High-Risk Pregnancy, Souissi Maternity Hospital, Rabat, MAR

**Keywords:** maternal morbidity, obstetric emergency, postpartum hemorrhage, puerperal hematoma, surgical management, vaginal delivery, vulvovaginal hematoma

## Abstract

Puerperal hematomas are rare but potentially life-threatening complications of vaginal delivery. They may expand rapidly, causing significant blood loss and hemodynamic instability. We report a case of a massive vulvovaginal hematoma occurring shortly after an apparently uncomplicated vaginal delivery in a 30-year-old multipara, presenting with severe perineal pain and signs of hypovolemia. Prompt surgical exploration with incision, drainage, and ligation of bleeding vessels resulted in complete recovery. This case highlights the importance of early clinical recognition and timely surgical management of puerperal vulvovaginal hematomas, even in the absence of classic risk factors.

## Introduction

Puerperal hematomas are defined as collections of blood within the connective tissue of the genital tract arising from vascular injury during childbirth. Although uncommon, with a reported incidence of one in 300 to one in 1500 deliveries, they represent a clinically significant cause of postpartum morbidity and, in severe cases, maternal mortality [1,2].

These hematomas are classified anatomically into vulvar, vaginal (paravaginal), and retroperitoneal subtypes. Vulvovaginal hematomas are the most frequently encountered and typically result from injury to branches of the internal pudendal artery. The retroperitoneal variety, though rare, carries the highest risk of massive concealed hemorrhage due to the absence of tamponade by surrounding tissue [3,4].

Recognized risk factors include primiparity, instrumental delivery (forceps or vacuum), median or mediolateral episiotomy, prolonged second stage of labor, macrosomia, and underlying coagulopathies [5]. Nonetheless, puerperal hematomas may occur in the absence of any identifiable predisposing factors, underscoring the need for heightened postpartum clinical vigilance in all patients [6].

The clinical hallmark is severe perineal pain disproportionate to examination findings, which may precede visible swelling. Without timely recognition and management, hematomas can expand rapidly, leading to hypovolemic shock, urinary retention, and infection [7]. We herein report a case of massive vulvovaginal hematoma managed surgically with excellent outcome, and review the current evidence on diagnosis and treatment.

## Case presentation

A 30-year-old woman (gravida 2, para 1) presented to the obstetric ward within hours following a vaginal delivery at term. The delivery had been documented as uncomplicated with no instrumentation. The newborn was appropriately grown, and placental delivery was complete with no immediate hemorrhagic concerns.

Within the first two hours postpartum, the patient developed intense, rapidly worsening perineal pain rated 9/10 on the numerical pain scale, associated with progressive vulvar swelling. She subsequently exhibited signs of hemodynamic compromise including tachycardia (heart rate 118 bpm) and hypotension (blood pressure 88/56 mmHg).

**Figure 1:**
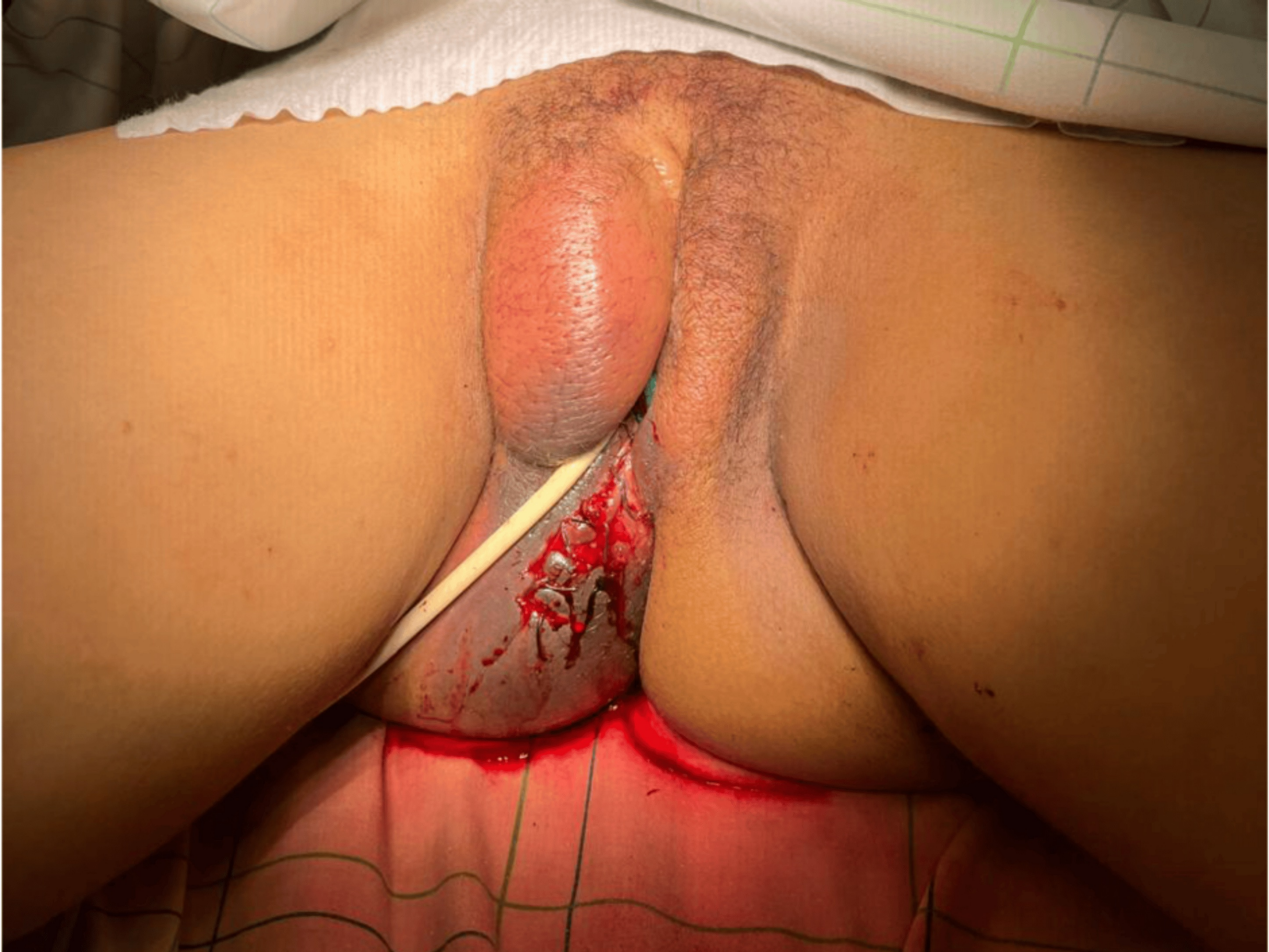
Intraoperative appearance showing a large, tense vulvovaginal hematoma predominantly on the right side, with ecchymosis and significant swelling prior to surgical incision.

Physical examination revealed a large, tense, bluish vulvovaginal mass predominantly involving the right labium majus and extending toward the posterior fourchette, measuring approximately 10 cm in its greatest dimension. The overlying skin demonstrated marked ecchymosis and exquisite tenderness on palpation (Figure 1).

Laboratory investigations revealed a hemoglobin level of 9.0 g/dL, decreased from an antenatal baseline of 11.8 g/dL. Coagulation profile (prothrombin time (PT), activated partial thromboplastin time (aPPT), fibrinogen) and platelet count were within normal limits. Bedside ultrasonography confirmed a heterogeneous collection consistent with a hematoma without intraperitoneal extension.

**Figure 2:**
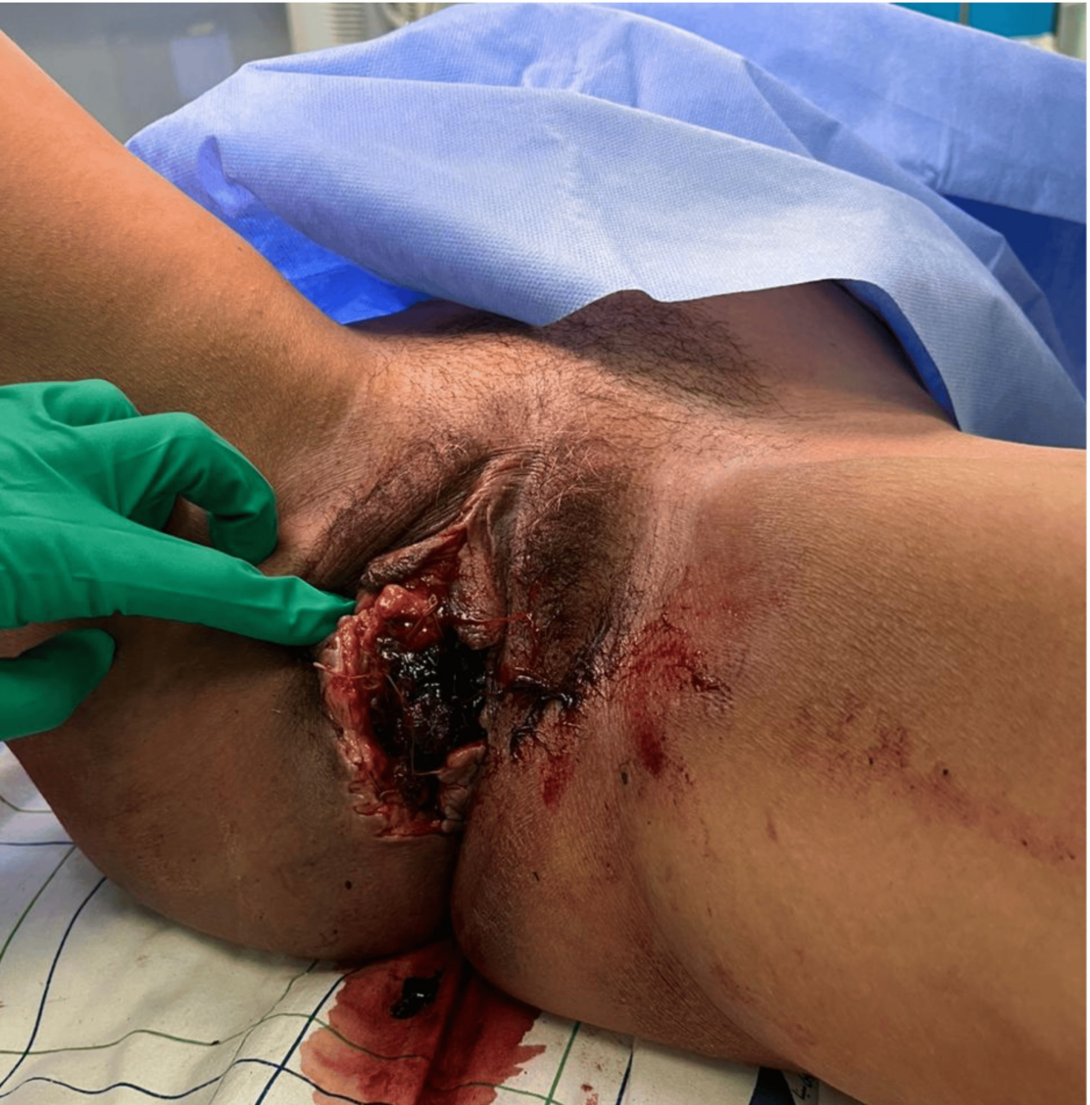
Intraoperative view following incision and drainage, demonstrating evacuation of approximately 200 mL of clotted blood and identification of an actively bleeding arterial vessel, consistent with a branch of the internal pudendal artery.

**Figure 3:**
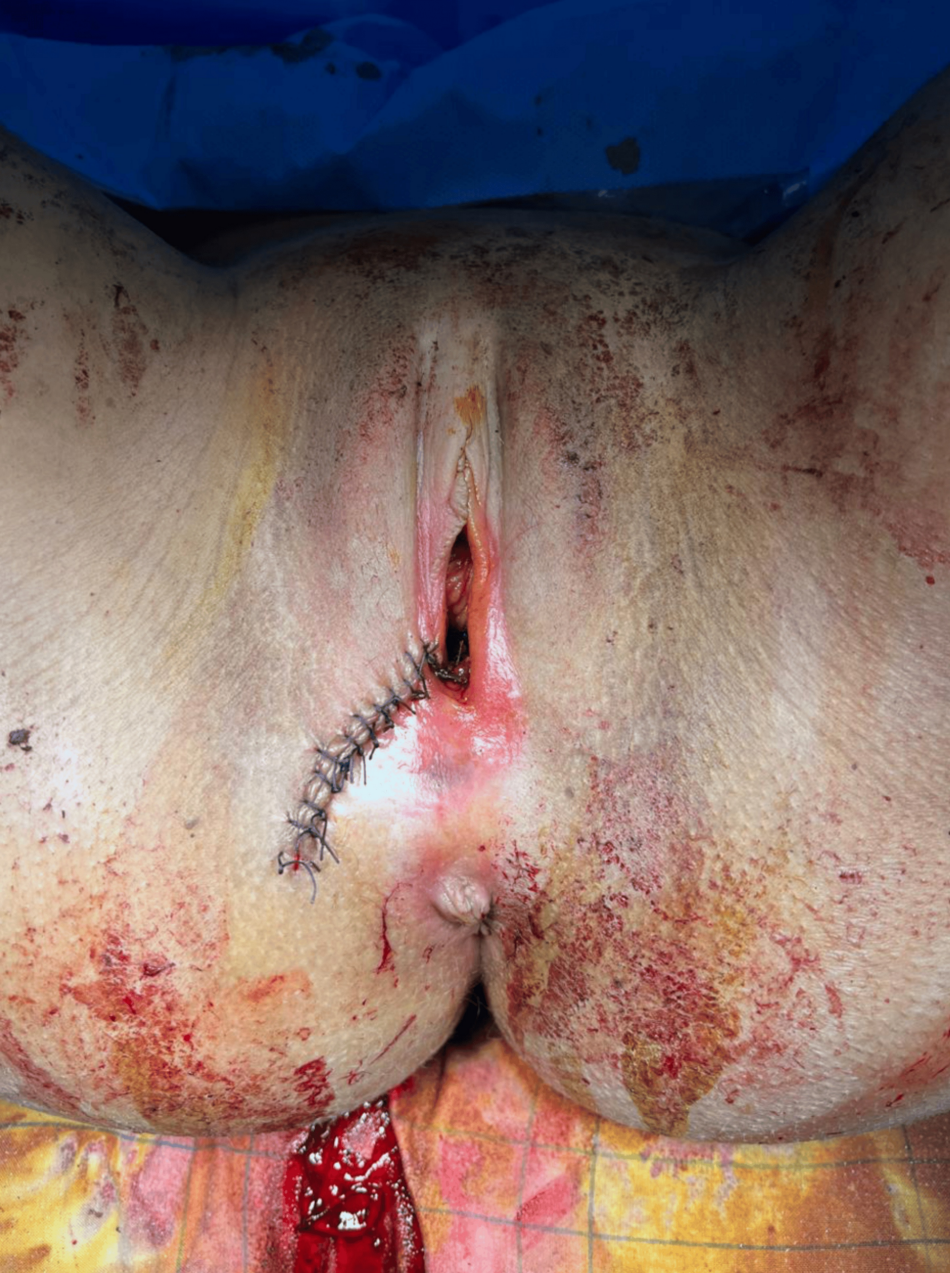
Postoperative appearance following hemostatic suturing, irrigation. The perineum shows satisfactory hemostasis with interrupted sutures and no residual hematoma bulk.

Given the rapid clinical deterioration and size of the hematoma, the decision was made to proceed with urgent surgical exploration under regional anesthesia. Intraoperative findings are illustrated in Figures 2, 3.

A longitudinal incision was made over the point of maximal fluctuance. Approximately 200 mL of clotted blood was evacuated. An actively bleeding arterial vessel, consistent with a branch of the internal pudendal artery, was identified and ligated with 2-0 Vicryl sutures. 

Postoperative management included intravenous fluid resuscitation, transfusion of two units of packed red blood cells, intravenous broad-spectrum antibiotic therapy (amoxicillin-clavulanate), regular hemoglobin monitoring, and analgesia with paracetamol and tramadol.

The postoperative course was uneventful. Vital signs stabilized within six hours. Follow-up hemoglobin at 48 hours was 10.2 g/dL. The drain was removed on postoperative day two, and the patient was discharged home in good clinical condition on postoperative day four, with outpatient wound follow-up arranged.

## Discussion

Puerperal genital hematomas are classified based on anatomical location into: (i) vulvar hematomas, confined to the labia majora or minora and bounded by the superficial fascia; (ii) paravaginal hematomas, arising in the paracolpium and potentially extending into the ischiorectal fossa; and (iii) retroperitoneal (paracervical or broad ligament) hematomas, which may reach considerable size before clinical detection [2,8].

The predominant blood supply to the vulvovaginal region is provided by the internal pudendal artery and its branches (inferior rectal, perineal, and posterior labial arteries). Tearing or avulsion of these vessels, with failure of adequate hemostasis, leads to progressive hematoma formation within the loose connective tissue of the vulvovaginal space [3,9].

The diagnostic hallmark is severe perineal pain that appears disproportionate to the degree of visible injury or to any laceration identified at delivery [7]. Other presentations include rapid vulvar or vaginal swelling, a palpable fluctuant mass, urinary retention secondary to urethral compression, and, in large hematomas, signs of hypovolemic shock.

Diagnosis is primarily clinical. Ultrasonography can confirm the diagnosis and delineate extension, particularly in cases where intra-abdominal spread is suspected. Computed tomography (CT) and magnetic resonance imaging (MRI) are reserved for complex or retroperitoneal cases [10]. Selective angiography may be used both diagnostically and therapeutically in cases of persistent or recurrent bleeding. Management strategy is determined by hematoma size, rate of expansion, and hemodynamic status of the patient 

Conservative management is appropriate for small (<3-5 cm), non-expanding hematomas in hemodynamically stable patients and consists of analgesia, ice application, bed rest, and close hemoglobin monitoring every four to six hours [4,11].

Surgical intervention remains the gold standard for large (>5 cm), rapidly expanding, or symptomatic hematomas, and in patients with hemodynamic instability [6,12]. The procedure involves a longitudinal or elliptical incision over the hematoma, complete evacuation of clot, identification and ligation or suture of bleeding vessels, copious irrigation, and closure over a drain to prevent reaccumulation.

Selective arterial embolization (SAE) of the internal pudendal or uterine artery is a minimally invasive alternative increasingly employed for cases of persistent or recurrent hemorrhage, particularly when bleeding vessels cannot be identified surgically, or in patients wishing to preserve fertility in the context of concomitant uterine hemorrhage [8,13]. Success rates exceeding 90% have been reported.

If not treated in a timely fashion, puerperal hematomas can lead to serious complications including hemorrhagic shock requiring massive transfusion, secondary infection with abscess formation, tissue necrosis, urinary tract complications, and prolonged hospitalization [7]. Rare cases of fatal outcome have been reported in low-resource settings. With appropriate and prompt management, however, prognosis is generally favorable.

Our case is notable in that the patient had no classic risk factors for puerperal hematoma: delivery was spontaneous, without instrumentation or episiotomy, and fetal weight was appropriate for gestational age. This emphasizes that puerperal hematoma may arise in any postpartum patient and that routine postpartum surveillance must include systematic perineal assessment, with particular attention to disproportionate pain.

## Conclusions

Puerperal vulvovaginal hematomas, while uncommon, represent a potentially life-threatening obstetric emergency. Severe postpartum perineal pain - particularly when disproportionate to visible findings - must prompt immediate perineal examination and hematoma exclusion. When a large or expanding hematoma is identified, prompt surgical management with incision, and vessel ligation is essential. Uterine or pudendal artery embolization is a valuable adjunct in refractory cases. The present case reinforces the importance of clinical vigilance, standardized postpartum assessment protocols, and rapid multidisciplinary response in achieving favorable maternal outcomes.
